# No improvement in vitamin D status in German infants and adolescents between 2009 and 2014 despite public recommendations to increase vitamin D intake in 2012

**DOI:** 10.1007/s00394-018-1717-y

**Published:** 2018-05-18

**Authors:** Clemens Kunz, Jürgen Hower, Anette Knoll, Kristin L. Ritzenthaler, Thomas Lamberti

**Affiliations:** 1 Institute of Nutritional Sciences, Justus Liebig University Giessen, Wilhelmstr. 20, 35392 Giessen, Germany; 2 Pediatric Group Practice KIDS 4.0, Melllinghoferstr. 256, 45475 Mülheim, Germany; 3 AK Statistics, Kreppe 2, 85276 Pfaffenhofen, Germany; 4 HiPP Werk Georg Hipp OHG, Georg-Hipp-Straße 7, 85276 Pfaffenhofen, Germany

**Keywords:** Vitamin D deficiency, Prevalence, German infants, Children, Adolescents

## Abstract

**Purpose:**

Vitamin D is a key component for the growth and development of children and adolescents, influencing a multitude of functions. Worldwide epidemiological studies have shown that minimum vitamin D blood levels of ≥ 20.0 ng/ml, often defined as vitamin D sufficiency by international and national nutrition and pediatric organizations, are often not met in practice. In 2012 the D–A–CH (Germany, Austria, Switzerland) nutrition societies increased their vitamin D intake recommendations fourfold from 200 IU (5 µg) to 800 IU (20 µg) per day. The outcome of this study will contribute to answering the question as to whether the new recommendations for increased vitamin D intake improve the highly prevalent vitamin D deficiency status in German children and adolescents.

**Methods:**

For this 6-year study (January 2009–December 2014) carried out in Mülheim an der Ruhr, Germany, healthy children and adolescents (*n* = 1929, age range 1–17 years, median age 11.0 years, 46.9% female) consulting a pediatric group practice (KIDS4.0) were recruited. Serum 25(OH)D determinations were performed using a competitive chemoluminescence immunoassay (CLIA, DiaSorin).

**Results:**

The median serum vitamin D values for each year from 2009 to 2014 were 18.4, 13.0, 20.8, 16.4, 19.4 and 14.9 ng/ml. The summarized median 25(OH)D serum concentrations between the two time periods 2009–2012 and 2013–2014 after increasing recommendations for vitamin D intake did not show a significant difference (17.0 versus 16.8 ng/ml).

**Conclusions:**

The increased D–A–CH recommendations for vitamin D intake had no influence on vitamin D levels in children and adolescents. The prevalence of vitamin D deficiency has not changed compared to previous studies.

**Electronic supplementary material:**

The online version of this article (10.1007/s00394-018-1717-y) contains supplementary material, which is available to authorized users.

## Introduction

The last decade has witnessed a tremendous interest in vitamin D, based on the awareness of a worldwide high prevalence of vitamin D deficiency. Particularly high-risk groups, such as infants, children, pregnant women and lactating mothers, elderly people, or individuals who cover their bodies for cultural reasons, are affected [1–8]. Vitamin D, long known for being essential in building and maintaining the integrity of the skeleton, has only in the last decade been recognized for its involvement in multiple other functions. Vitamin D modulates, regulates, and stabilizes the immune and defense system [9–11]. It may therefore not be surprising that vitamin D deficiency has been linked to a multitude of negative health outcomes such as cancer, auto-immune diseases, infections, allergies, asthma and even depression [12–15]. These new insights have led public health authorities and nutritional societies to reconsider their recommendations for daily vitamin D intake [16, 17]. In January 2012, the nutritional societies of Germany, Austria and Switzerland (D–A–CH) increased their vitamin D intake recommendations for all age groups [18]. In 2013, the European Society for Pediatric Gastroenterology, Hepatology and Nutrition (ESPGHAN) drew attention to the problem of vitamin D deficiency among European children and adolescents, and called for action [19]. There is an urgent need for strategies to improve the vitamin D status of children and adolescents, which has recently been reconfirmed by a consortium of European pediatricians [20]. Although many national and international authorities recognize the need for improvement, they seem to fail in translating knowledge into current practice. The D–A–CH nutrition societies only recommend vitamin D supplementation if there is no endogenous production via solar exposure. This means, in practice, that children, adolescents, parents and primary caregivers are left alone in evaluating the effectiveness of their solar exposure and/or their vitamin D status through their 25(OH)D blood levels.

The question as to whether a recommended higher vitamin D intake would reduce the prevalence of vitamin D deficiency in our most vulnerable population group, in children and adolescents, needs to be answered. The main aim of our study was (1) to evaluate the vitamin D status of various age groups from the first years until adulthood and (2) to compare the 25(OH)D status in period 1 (years 2009–2012) with that in period 2 (years 2013–2014), reflecting the vitamin D status of children and adolescents before and after the fourfold increase in D–A–CH recommendations (from 200 to 800 IU per day).

## Methods

### Study population

This was a non-interventional study of serum 25(OH)D data samples to gain further insight into vitamin D status under regular patient settings. The study participants were patients who visited a pediatric medical group practice in Mülheim an der Ruhr (Germany 51°N) for various reasons. Access to healthcare in Germany is very easy and many preventive services are provided. 75% of the study participants were of German or European (Caucasian) origin. The remaining 25% had mostly Turkish roots, 2% showed a worldwide distribution (Africa and Asia). An advice for taking supplements was not given. German guidelines recommend vitamin D only for the first 12–18 months of life depending on the time of birth (if born in winter, then 18 months are recommended). Questions regarding the level of activity or dietary behavior were not asked. However, there was no correlation with solar exposure in Mülheim (data are available but not shown here) which indicates that outdoor activity had no impact on 25(OH)D. All children were tested only once with the exception of 2013 and 2014. In those two years 24 patients provided a blood sample in 2013 as well as in 2014. In total 1957 samples have been analyzed from 1909 participants.

### Biochemical analysis

Blood was taken from healthy patients who mainly came for a check-up. Some patients mentioned minor functional complaints (occasional headaches, attention problems, muscle aches, and others) in which major diseases could be excluded. Fasting venous blood samples were drawn in the morning and sent to the laboratory the same day. Blood analyses were performed by a central laboratory (Bioscientia GmbH, Ingelheim, Germany). Quantitative analysis of 25(OH)D in serum was done by means of direct competitive chemoluminescence immunoassay (CLIA, DiaSorin). 25(OH)D concentrations under the lower limit of quantification (LLOQ) were set to 0.5*LLOQ. Details have been described elsewhere [21].

### Data assessment and statistics

The data base was anonymized before analysis. Study approval was obtained from the Ethics Committee of the Medical Faculty, Justus-Liebig University Giessen. The data were divided into period 1 (2009–2012) and period 2 (2013–2014) to account for the recommendations in vitamin D increase by the nutrition societies of Germany, Austria and Switzerland in 2012 [18].

Vitamin D levels were grouped into four classification stages, i.e., (1) < 10 ng/ml severe deficiency; (2) 10 to < 20 ng/ml deficiency; (3) 20 to < 30 ng/ml sufficiency and (4) ≥ 30–80 ng/ml physiological status. This classification is based on a combination of recommendations by both, the DKGJ and ESPGHAN (which mainly follow the IOM) [19, 22, 23] as well as on the opinion of the Endocrine Society [24] and authors of the HELENA study [25]. According to DGKJ and ESPGHAN, a 25(OH)D ≤ 20 ng/ml indicates vitamin D deficiency. If the 25(OH)D concentration was between a deficiency and a *“*physiological*”* status we would consider this a sufficiency status (> 20–30 ng/ml), meaning that in most cases clinical symptoms can be avoided at such concentrations. The Endocrine Society and the HELENA study argue that for skeletal and non-skeletal effects 30 or even 40 ng/ml would be optimal. In our study we use “physiological status” instead of “optimal status” for the following reasons: (1) “physiological” 25 OH D concentration can be considered as those which are measurable in individuals with a natural vitamin D skin production as it is well accepted that a vitamin D intoxication through sun exposure is not possible [26–28]. Through sun exposure levels of 80 ng/ml 25 OH D can be reached with no effects on other parameter regarding calcium and phosphate homeostasis [29–32]. Hence, those concentrations can be regarded as “physiological”. In addition, skin vitamin D production is very efficient which, under appropriate conditions, can lead to 10,000–25,000 IU within a few minutes without reaching higher levels than 80 ng/ml [30, 33].

To investigate the seasonal influence on serum 25(OH)D levels, samples were grouped according to their date of collection into spring (March–May), summer (June–August), fall (September–November) and winter (December–February), as reported earlier [34].

Descriptive statistics have been applied for analyzing continuous parameters; categorical variables were presented with counts and percentages. Subgroup analyses were conducted with respect to gender, age groups, vitamin D classification stages, year and season. Inferential analyses were carried out using non-parametric (Wilcoxon two-sample test) or multivariate analyses of variance methods (ANOVA based on ranks) to investigate age group, year and treatment period-specific differences in absolute vitamin D levels. For ANOVA models, varying combinations of fixed variables were included depending on the hypothesis to be investigated. Relevant confounders comprised year, gender, season and treatment periods. Inferential analyses of patients being in different vitamin D classification stages were carried out using the Chi square test. All tests were executed at an explorative significance level of 5% (two-sided). Data analysis was carried out using SAS version 9.3 (SAS Institute Inc., Cary, NC, USA).

## Results

### Overall vitamin D status from 2009 to 2014 and % distribution in the four classification stages

The vitamin D status was analyzed retrospectively in 1957 blood samples collected from 1909 children and adolescents (age 0–17 years) between 2009 and 2014. The reason for the difference between the number of blood samples and children is that 24 patients provided a blood sample in 2013 as well as in 2014. Table 1 shows characteristics of the sample cohort. For comparative reasons, Table 1 also lists characteristics for the combined years 2009–2012 (period 1) and 2013–2014 (period 2). Although the number of analyzed samples steadily increased from 2009 to 2014, total number of samples in period 1 (2009–2012) and period 2 (2013–2014) were similar (943 versus 1014, Table 1). The median (interquartile range) age of the total population was 11.0 (7.0, 13.0) years with the majority of samples (27% each) originating from the age groups 7–10 and 11–13.

**Table 1:** Demographic characteristics of sample set

	2009	2010	2011	2012	2013	2014	2009–2012	2013–2014	Total
No. of samples (*n*, %)	109 (5.6)	195 (10.0)	222 (11.3)	417 (21.3)	447 (22.8)	567 (29.0)	943 (48.2)	1014 (51.8)	1957^a^
No. of patients	109	195	222	417	447	567	943	967	1909^b^
Gender (*n*, %)									
Male	59 (54.1)	107 (54.9)	114 (51.4)	221 (53.0)	251 (56.2)	287 (50.6)	501 (53.1)	538 (53.2)	1039 (53.1)
Female	50 (45.9)	88 (45.1)	108 (48.6)	196 (47.0)	196 (43.8)	280 (49.4)	442 (46.9)	476 (47.8)	918 (46.9)
Age^c^ (years)	7.0 (3.0, 10.0)	9.0 (4.0, 11.0)	10.0 (7.0, 13.0)	11.0 (8.0, 14.0)	12.0 (8.0, 14.0)	11.0 (7.0, 13.0)	10.0 (6.0, 13.0)	11.0 (7.0, 14.0)	11.0 (7.0, 13.0)
Age groups (years) (*n*, %)									
0–2	20 (18.4)	30 (15.2)	16 (7.2)	15 (3.6)	12 (2.7)	33 (5.8)	81 (8.6)	45 (4.4)	126 (6.4)
0–12 months	7 (6.4)	9 (4.6)	2 (0.9)	2 (0.5)	0 (0.0)	4 (0.7)	20 (2.1)	4 (0.4)	24 (1.2)
> 12–24 months	13 (12.0)	21 (10.8)	14 (6.3)	13 (3.1)	12 (2.7)	29 (5.1)	61 (6.5)	41 (4.0)	102 (5.21)
3–6	31 (28.4)	51 (26.2)	39 (17.6)	55 (13.2)	64 (14.3)	87 (15.3)	176 (18.7)	151 (14.9)	327 (16.7)
7–10	33 (30.3)	55 (28.2)	64 (28.8)	123 (29.5)	88 (19.7)	157 (27.7)	275 (29.2)	245 (24.2)	520 (26.6)
11–13	15 (13.8)	35 (18.0)	66 (29.7)	101 (24.2)	150 (33.6)	161 (28.4)	217 (23.0)	311 (30.7)	528 (27.0)
14–17	10 (9.2)	24 (12.3)	37 (16.7)	123 (29.5)	133 (29.8)	129 (22.8)	194 (20.6)	262 (25.8)	456 (23.3)
Season^d^ (*n*, %)									
Spring	21 (19.2)	75 (38.4)	48 (21.6)	127 (30.5)	118 (26.4)	209 (36.9)	271 (28.7)	327 (32.2)	598 (30.6)
Summer	39 (35.8)	25 (12.8)	43 (19.4)	91 (21.8)	143 (32.0)	116 (20.5)	198 (21.0)	259 (25.6)	457 (23.4)
Fall	26 (23.9)	60 (30.8)	64 (28.8)	123 (29.5)	103 (23.0)	116 (20.5)	273 (29.0)	219 (21.6)	492 (25.0)
Winter	23 (21.1)	35 (18.0)	67 (30.2)	76 (18.2)	83 (18.6)	126 (22.1)	201 (21.3)	209 (20.6)	410 (21.0)

^a, b^Difference between total number of patients and total number of samples: 24 patients provided a blood sample in 2013 as well as in 2014

^c^Median (interquartile range)

^d^Seasons were classified into spring (March–May), summer (June–August), fall (September–November) and winter (December–February)

**Fig. 1:**
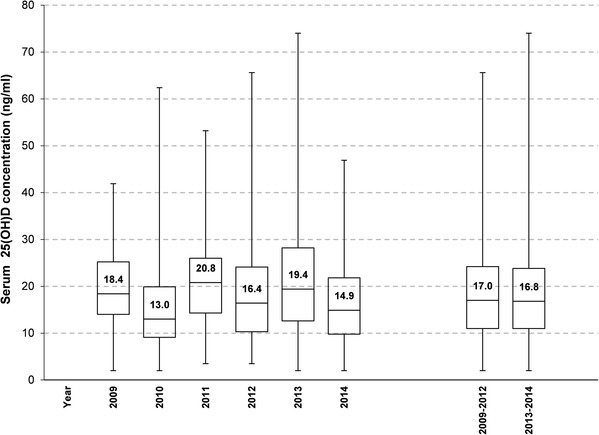
Overall vitamin D status in each year. The data are presented as box plots with median and interquartile range [IQR (Q3–Q1), whiskers indicate minimum and maximum]. ANOVA based on rank models adjusted for season and age group revealed statistically significant differences in all years except between 2009 and 2011, 2009 and 2013, 2011 and 2013, 2012 and 2014, and between periods 2009–2012 and 2013–2014

The median 25(OH)D levels of the total study population and the interquartile ranges in each year are shown in Fig. 1. This data, which gives a first indication of vitamin D status, shows that there were large individual variations with a median serum 25(OH)D usually below 20 ng/ml (= 50 nmol/l). Although individual years show significant differences in their median vitamin D status, this difference disappears when comparing the time periods before and after the updated vitamin D intake recommendation (2009–2012 and 2013–2014; 17.0 versus 16.8 ng/ml).

**Fig. 2:**
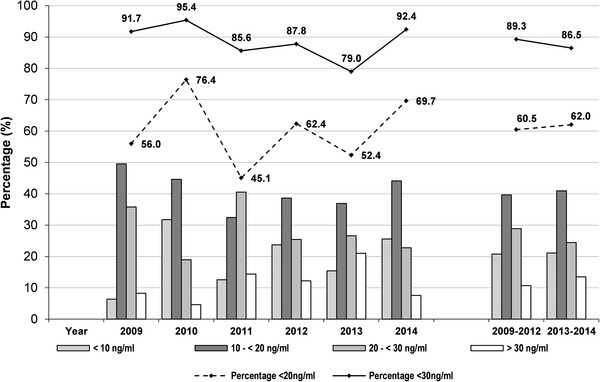
25(OH)D distribution (%) in each year according to the four different vitamin D classification stages. In addition, the percentage of samples not reaching 25(OH)D levels ≥ 20 ng/ml or ≥ 30 ng/ml (upper part) is shown

In Fig. 2, the vitamin D status for each year is graphically presented according to the four classification stages described in “Data Assessment and Statistics”. The cumulative percentage of samples per year with 25(OH)D concentrations below 20 ng/ml or below 30 ng/ml is shown in the upper section. Comparing each year from 2009 to 2014, between 45 and 76% of all participants had 25(OH)D concentrations below 20 ng/ml, 79–95% of serum 25(OH)D was below 30 ng/ml. More detailed information is given in Supplemental Table 1. The most prominent year with a severe vitamin D deficiency with 25(OH)D below 10 ng/ml was 2010, with more than 30% of all subjects in this class. Data in the other years varied between 6.4 and 25.6%. A statistical comparison of the combined data between period 1 and period 2 did not show any significant difference.

### Vitamin D status in different age groups

**Fig. 3:**
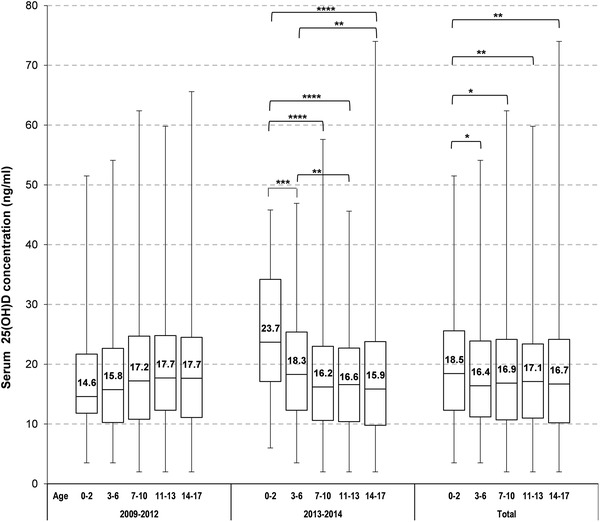
Overall vitamin D status for each age group and for periods 1 and 2. Data are presented as box plots with median and interquartile range [IQR (Q3–Q1), whiskers indicate minimum and maximum]. Differences between age groups within each period and overall (2009–2014) tested with ANOVAs based on ranks adjusted for year and season (*p* value two-sided): *p < 0.05, **p < 0.01, ***p < 0.001, ****p < 0.0001

To differentiate between the various age groups the median and interquartile ranges in period 1 and 2 are given in Fig. 3. Solely for infants up to 2 years of age in 2013–2014 the median values reached levels above 20 ng/ml. The sufficiently high 25(OH)D concentration in this age group may be influenced by the low number of samples in 2013–2014 (*n* = 45) compared to *n* = 81 in 2009–2012 (Table 2). A significant difference between period 1 and 2 was also found in the age group 3–6 years (median 15.8 ng/ml (IQR [10.3, 22.7]) in period 1 and 18.3 mg/ml (IQR [12.3, 25.4]) in period 2; *p* = 0.035, Table 2). Although a significant difference could be confirmed in the age group of 11–13 year-old adolescents, the effect was inverse with a lower 25(OH)D median after the increase of the vitamin D recommendations. Overall, in the various age groups the proportion of samples with 25(OH)D concentration < 20 ng/ml is still high with more than half of the children and adolescents revealing (severe) vitamin D deficiency (0–2 years 54.0%, 3–6 years 62.7%, 7–10 years 61.1%, 11–13 years 61.6% and 17–18 years 62.0%) (Suppl. Table 2).

**Table 2:** Gender and age-specific serum 25(OH)D characteristics before and after vitamin D recommendation by the nutrition societies of Germany, Austria and Switzerland (D–A–CH 2012)

Year	Age group (years)	Male	Female	Total	*p* value^a^	*p* value^b^
2009–2012	0–2					
	*n*	39	42	81		
	Mean ± SD	17.2 ± 10.95	17.4 ± 6.90	17.3 ± 9.02		
	Median (interquartile range)	14.0 (9.4, 22.0)	16.1 (13.2, 21.7)	14.6 (11.8, 21.7)	0.3028	
	3–6					
	*n*	95	81	176		
	Mean ± SD	17.2 ± 9.31	17.0 ± 8.37	17.1 ± 8.67		
	Median (interquartile range)	16.0 (10.0, 22.9)	15.2 (10.4, 21.8)	15.8 (10.3, 22.7)	0.9444	
	7–10					
	*n*	154	121	275		
	Mean ± SD	18.9 ± 10.16	18.4 ± 10.39	18.6 ± 10.24		
	Median (interquartile range)	17.5 (10.9, 24.5)	17.0 (10.3, 24.7)	17.2 (10.8, 24.7)	0.7054	
	11–13					
	*n*	117	100	217		
	Mean ± SD	19.0 ± 8.97	19.1 ± 10.58	19.0 ± 9.72		
	Median (interquartile range)	18.0 (12.9, 25.0)	17.6 (12.0, 24.6)	17.7 (12.3, 24.8)	0.6550	
	14–17					
	*n*	96	98	194		
	Mean ± SD	18.1 ± 9.74	18.5 ± 9.36	18.3 ± 9.53		
	Median (interquartile range)	17.2 (10.6, 24.4)	18.4 (11.1, 24.6)	17.7 (11.1, 24.5)	0.6553	
2013–2014	0–2					
	*n*	26	19	45		
	Mean ± SD	26.3 ± 10.12	23.1 ± 10.08	25.0 ± 10.12		
	Median (interquartile range)	24.9 (18.5, 35.6)	22.0 (16.5, 32.4)	23.7 (17.1, 34.2)	0.3520	< 0.0001
	3–6					
	*n*	82	69	151		
	Mean ± SD	20.6 ± 10.04	18.3 ± 8.60	19.6 ± 9.45		
	Median (interquartile range)	18.5 (12.6, 26.8)	17.3 (10.5, 23.8)	18.3 (12.3, 25.4)	0.2507	0.0356
	7–10					
	*n*	137	108	245		
	Mean ± SD	18.6 ± 9.91	17.6 ± 10.35	18.2 ± 10.10		
	Median (interquartile range)	16.8 (10.8, 23.8)	15.6 (10.0, 22.7)	16.2 (10.6, 23.0)	0.3428	0.2977
	11–13					
	*n*	164	147	311		
	Mean ± SD	18.7 ± 8.89	15.7 ± 9.06	17.3 ± 9.08		
	Median (interquartile range)	18.3 (11.7, 23.6)	13.7 (8.6, 21.8)	16.6 (10.4, 22.7)	0.0025	0.0472^c^
	14–17					
	*n*	129	133	262		
	Mean ± SD	17.3 ± 9.91	18.9 ± 11.87	18.1 ± 10.95		
	Median (interquartile range)	15.5 (9.6, 23.8)	16.5 (10.6, 23.6)	15.9 (9.8, 23.8)	0.4444	0.3898

^a^Two-sided *p* values derived from Wilcoxon two-sample test evaluating age-specific differences in serum 25(OH)D concentrations between female and male subjects

^b^Two-sided *p* values derived from ANOVA based on ranks including gender and season as fixed factors. ANOVA tested differences of total serum 25(OH)D concentrations between the years 2009–2012 and 2013–2014 within each age group. Season had a significance level of 5% in all models

^c^Significant, but median total serum 25(OH)D concentration of 2013–2014 in age group 11–13 years did not improve compared to 2009–2012. Gender appears as an additional significant factor

### Seasonal influence on vitamin D status

**Fig. 4:**
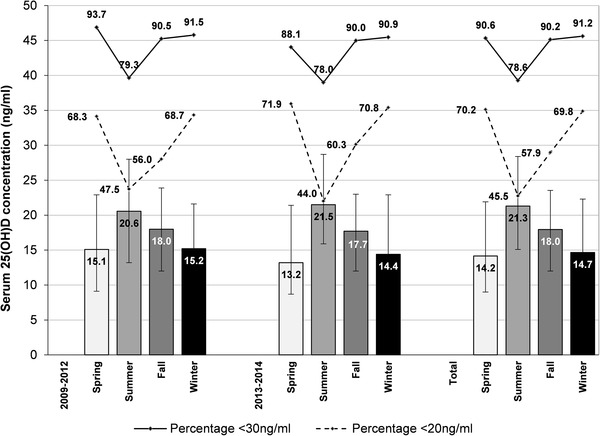
Seasonal influence on 25(OH)D values in period 1 (2009–2012) and period 2 (2013–2014). Data are presented as median and interquartile range [IQR (Q3–Q1)] and as percentage of samples classified as vitamin D deficient (< 20 ng/ml) or vitamin D insufficient (< 30 ng/ml)

The data show clear seasonal variations according to the month of sample collection. The highest overall median 25(OH)D levels were found in summer (Fig. 4, on the right; 21.3 ng/ml), followed by fall (18.0 ng/ml), winter (14.7 ng/ml) and spring (14.2 ng/ml). In the total study population, close to one-third of all spring samples and one-fourth of all winter samples were classified as severe vitamin D deficient (< 10 ng/ml), decreasing to 10% during summer months.

In period 1 (2009–2012), the percentage of samples with a 25(OH)D level below 20 ng/ml was 47.5% in summer, 56.0% in fall, 68.7% in winter and 68.3% in spring (Fig. 4, left); in period 2 (2013–2014), 44.0% (summer), 60.3% (fall), 70.8% (winter) and 71.9% (spring) of the samples did not reach 20 ng/ml.

### Vitamin D status according to gender and age

Gender and age-specific 25(OH)D data are shown in Table 2. In period 1 (2009–2012), the median for males varied between 14.0 ng/ml (0–2 years) and 18.0 ng/ml (11–13 years) and for females between 15.2 ng/ml (3–6 years) and 18.4 ng/ml (14–17 years). In period 2 (2013–2014), males aged 14–17 and females aged 11–13 years showed the lowest median concentration with 15.5 and 13.7 ng/ml, respectively. Table 2 also includes inferential comparisons of 25(OH)D levels between males and females in both time periods, as well as comparisons of total 25(OH)D levels between the two periods. We did not find any significant gender-specific differences in any age class neither in period 1 (2009–2012) nor in period 2 (2013–2014) with the exception of the age classes 0–2, 3–6 and 11–13 in period 2013–2014, which revealed a gender effect with higher 25(OH)D levels in males compared to females and a significant difference in total 25(OH)D levels between the two periods.

## Discussion

In 2012, Germany, Austria, and Switzerland increased their vitamin D intake recommendations twofold for toddlers, and even fourfold for children and adolescents, in comparison to previous recommendations [from 5 µg (200 IU) vitamin D to 20 µg (800 IU) per day] [18] (D–A–CH 2012). These changes were based on the increasing awareness of the high prevalence of vitamin D deficiency influencing growth and development in children and adolescents [1, 2, 4, 7, 8, 11, 13, 19, 35–39].

The issue of which concentration of 25(OH)D in blood best reflects an appropriate vitamin D status is still a matter of controversy [5, 17, 22, 24, 40, 41]. The German Society of Pediatrics and Adolescent Medicine (DGKJ) [23] defined “vitamin D deficiency” at 25(OH)D concentrations in serum below 20 ng/ml. Concentrations < 10 ng/ml were classified by ESPGHAN  as “severe vitamin D deficiency” [19]. For high-risk groups, an adequate vitamin D supply is recommended to guarantee 25(OH)D concentrations of 30 ng/ml and more [19]. The latter is in agreement with the US Endocrine Society [42] which defined the “sufficiency status” at serum 25(OH)D concentrations of at least 30 ng/ml based on data showing a minimal PTH release and maximum calcium absorption [42, 43].

We used a combination of recommendations, i.e., a “deficiency” cut-off 25(OH)D ≤ 20 ng/ml given by the DKGJ [23] and ESPGHAN [19] (which mainly follow the IOM [22]) as well as the opinion of the Endocrine Society [33] and authors of the HELENA study [24] with regard to an “optimal” 25(OH)D status (≥ 30 ng/ml) to evaluate whether the increased recommendations for vitamin D in Germany led to an improved vitamin D status in the following years. The results represent the first data on 25(OH)D serum levels of infants, children and adolescents of the KIDS 4.0 study. Blood was collected in a pediatric group practice in Mülheim an der Ruhr between 2009 and 2014. The median serum 25(OH)D level during the study period ranged between 13.0 and 20.8 ng/ml with large individual variations. According to the current statements of ESPGHAN and other societies, this data indicates a vitamin D deficiency status [25(OH)D < 20 ng/ml] of most children and adolescents throughout all seasons. Even during the summer months, median 25(OH)D concentrations were low [20.6 ng/ml in period 1 (2009–2012) and 21.5 ng/ml in period 2 (2013–2014)]. This may be partly motivated by an indoor lifestyle and sun protection measures. Due to decreasing solar exposure, vitamin D serum levels fall during winter and reach their nadir in early spring. Even 25(OH)D levels indicating a severe vitamin D deficiency according to the ESPGHAN criteria [25(OH)D lower than 10 ng/ml] were found in 6–32% of the study participants. This study confirms our previous results from a small pilot study in 2007/2008, with 202 participating children and adolescents from the same urban area [21].

When classifying our data according to age groups, median 25(OH)D serum levels reached little more than 20 ng/ml only in infants aged 0–2 years in the years 2013–2014. This is most likely the result of the vitamin D supplementation which is recommended during the first 12–18 postnatal months in Germany. The fact that more than 50% of infants between 0 and 2 years in period 1 (2009–2012) did not reach vitamin D sufficiency status (median 14.6 ng/ml) despite regulatory advice for vitamin D prophylaxis (400–500 IU/D) may be explained by the small number of samples and possible selection bias.

Our observations in children and adolescents living in Germany support the conclusion that vitamin D serum levels in these age groups are even lower than in adults, as reported in the KiGGS study [44, 45] and recently in the DEGS1 study [46]. In the Nutrition and Lifestyle in European Adolescents (HELENA) study, which included only adolescents aged between 12.5 and 17.5 years from ten countries, about 42% of all participants had 25(OH)D concentrations below 50 nmol/l (or 20 ng/ml); the lowest 25(OH)D concentrations were measured in adolescents from Dortmund (Germany) with a mean level of 49 nmol/L [25]. In our cross-sectional study, we could show that the median vitamin D serum level in children and adolescents was mostly insufficient which is in agreement with the Helena study. Low 25(OH)D levels, in particular at puberty, have also been found in many European countries [47–51].

Although the health risk associated with an insufficient or deficient vitamin D status has been increasingly recognized, often confusing or misleading recommendations have been given to the public. In a recent publication, the authors recommended: “… efforts should be made to maintain vitamin D status throughout the year by spending time outside regularly (without taking risks of sunburns and skin cancer) and paying attention to a healthy diet rich in vitamin D, especially during winter and spring” [46]. This is a representative example for many publications, and official statements in various countries which, although recognizing the health risks resulting from vitamin D deficiency, recommend activities which are ineffective in their translation into practice for various reasons: (1) due to lifestyle changes, children and adolescents are no longer involved in enough outdoor activities, as many publications confirm; (2) in Germany, as well as in various other European countries, even if enough time is spent outdoors, it would be impossible to produce sufficient vitamin D in the skin during half of the year (roughly from October to March) as sufficient solar UVB radiation is missing [2, 52]; (3) it has been known for a long time (and is recalled by the same authorities in their statements) that dietary vitamin D intake provides only 5–10% of the recommendations, meaning that vitamin D dietary intake is negligible and vitamin D status cannot be improved by a healthy diet alone; (4) low storage of vitamin D in the liver and fat tissue are considered reasons for low 25(OH)D levels in winter and spring. If we take into account that body fat may store vitamin D, the question arises as to why obese individuals show a mean 35% and overweight individuals a mean 24% higher vitamin D deficiency status compared to average weight individuals, and why they must be considered as a high risk group for vitamin D deficiency [53].

Our study has some limitations which may have an influence on the vitamin D status. For example, (1) the vitamin D intake by diet or supplements is unknown; (2) low outdoor activity and frequent screen time (TV or computer) are relevant parameter that could explain low vitamin D supply. Since leisure activities are not considered in this study it cannot be concluded if low vitamin D supply, even in the summer months, can be explained either by the amount of outdoor activities or by the low solar radiation in Germany; (3) the color of the skin (skin type) was not considered but 75% of the subjects were of Caucasian origin; (4) body weight has not been recorded. This should be investigated in more detail in further studies as it is known that not only overweight but also underweight has to be considered as risk factor triggering low vitamin D supply and (5) social status and education of the subjects are not known.

The strength of our study is the collection of samples under everyday conditions in a pediatric group practice. A previous study on vitamin D in Mülheim (Germany) has shown that the data correspond very closely to the KiGGs results [54]. The current study is an additional and more detailed analysis of samples collected over many years showing the same results. Hence, we are convinced that the results can be generalized.

The data obtained correspond to the median vitamin D serum levels of the KiGGS study (10,015 boys and girls, 267 cities and communities, May 2004–May 2006, median vitamin serum level 16.96 ng/ml) [35]. The KIGGS study and our study are comparable as both covered the age between birth up to 17 years; as the KIGGS data have been collected from 2003 to 2006, long time before the recommendations have been increased by the DACH societies, we can conclude that all activities to improve the vitamin D status so far have been ineffective.

As the methods for determining vitamin D and metabolites is of particular importance we used the diagnostic RIA assay from DiaSorin which comes close to the mostly recommended gold standard LC-MS/MS (liquid chromatography–tandem mass spectrometry), but which is only available in a few places. In a comparison study, the RIA assay showed a performance comparable to the LC-MS/MS method with a concordance correlation coefficient of 0.97 [55]. Any differences due to the DiaSorin assay should therefore be negligible for the interpretation of our data set.

Considering the importance of an adequate vitamin D status for children and adolescents, we need to answer the question as to why efforts to improve the vitamin D status in children, adolescents and in the adult population have failed in spite of current guidelines. There is an urgent need to implement clear and practical strategies in order to translate scientific knowledge into practice. Our results show that just changing guidelines is not enough and has no bearing on vitamin D status.

The question as to what may be the best strategy to improve the population-wide deficient vitamin D status must be answered. We strongly recommend that public health authorities and nutrition societies should not only focus on recommending a higher vitamin D intake, but concentrate more on strategies to translate current knowledge and guidelines successfully into practice. It is the responsibility of scientific societies (1) to guarantee that the population can follow their recommendations and (2) to ensure that the health risk is kept at a minimum by finding ways to improve an insufficient vitamin D status. In Germany, as in many other European countries, dietary vitamin D intake is negligible and UVB-induced epidermal production too low or non-existent during about 6 months of the year. Therefore, the question needs to be answered as to whether supplementation or food fortification or a combination of both strategies are options to improve the status. Certainly, this is not an easy task, but increasing recommendations without such strategies seem to be ineffective, as shown by our results. Successful strategies to improve vitamin D status in Northern European children have been discussed [56]. The data show that, for example, food fortification is feasible to maintain adequate circulating vitamin D levels, especially during winter months.

Despite fortification of milk products and vegetable oils, EVIRA, the Finnish Food Safety Authority, started to recommend that all children, adolescents and adults should be supplemented with 10 µg of vitamin D per day throughout the entire year. It may be possible to also reach the target of vitamin sufficiency [minimum serum 25(OH)D level of 20 ng/ml] via sufficient intake of milk and fortified vegetable oil [57]. The British National Health Service (NHS) and Public Health England (PHE) also advise that all children over 1 year of age and adults should consider taking a daily supplement containing 10 µg of vitamin D, particularly during fall and winter [58].

We possess no information about the impact of these new recommendations in Finland and in Great Britain on vitamin D status. Our current data show that the results of the D–A–CH vitamin D guideline update in 2012 has had no effect on the vitamin D status of children and adolescents living in Mülheim an der Ruhr. We may cautiously assume that this holds true for all German children and adolescents. Other nutritional studies have also shown that it is difficult to follow recommendations in changing lifestyles to improve health-related behavior [59, 60]. Our results suggest, that respective societies should ponder on the reasons why these recommondations fail in improving the health status of their addressees. A lack of simplicity and clarity (being indispensable for the implementation into everyday life) is characteristic for the recommendations currently available, be it from ESPGHAN, DGKJ or D–A–CH. Often, there are mainly three reasons why guidelines are not followed by the general population: first, a lack of sufficient information with respect to significance and meaning of the published guidelines, second, the lack of clarity and simplicity of the guidelines, and third, both resulting in a lack of motivation to follow the guidelines [61].

## Conclusion

The results show, as a primary outcome, that the D–A–CH changes in vitamin D intake recommendations have had no influence on actual vitamin D levels in children and adolescents. The overall vitamin D status of children and adolescents in Mülheim an der Ruhr has not changed in comparison to previous studies. The questions are: where can we go from here and which strategy may improve vitamin D sufficiency? Perhaps food enrichment or vitamin D supplements, or both? Different approaches need to be tested to ensure the future health of the younger generation.

## Electronic supplementary material

Below is the link to the electronic supplementary material.

Supplementary material 1 (DOCX 15 KB)

Supplementary material 2 (DOCX 15 KB)
