# Supplementary material for: No improvement in vitamin D status in German infants and adolescents between 2009 and 2014 despite public recommendations to increase vitamin D intake in 2012
Source: Eur J Nutr. 2018 May 18;58(4):1711–22. doi: 10.1007/s00394-018-1717-y (PMC6561984; doi:10.1007/s00394-018-1717-y)
Supplement: Supplementary file 1 — Supplementary material 1 (DOCX 15 KB) [file 394_2018_1717_MOESM1_ESM.docx]

**Suppl. Table 1** Distribution of serum 25(OH)D levels in years 2009–2014 according to the four classification stages^1)^

|  |  |  |  | Year |  |  |  |  |  |
| --- | --- | --- | --- | --- | --- | --- | --- | --- | --- |
|  | 2009 | 2010 | 2011 | 2012 | 2013 | 2014 | 2009–2012 | 2013–2014 | Total |
| Subjects with |  |  |  | **n (%)** |  |  |  |  |  |
| **Deficiency (<20 ng/ml)** | **61 (56.0)** | **149 (76.4)** | **100 (45.1)** | **260 (62.4)** | **234 (52.4)** | **395 (69.7)** | **570 (60.5)** | **629 (62.0)** | **1199 (61.3)** |
| <10 ng/ml: Severe deficiency | 7 (6.4) | 62 (31.8) | 28 (12.6) | 99 (23.7) | 69 (15.4) | 145 (25.6) | 196 (20.8) | 214 (21.1) | 410 (21.0) |
| 10-<20 ng/ml: Deficiency | 54 (49.5) | 87 (44.6) | 72 (32.4) | 161 (38.6) | 165 (36.9) | 250 (44.1) | 374 (39.7) | 415 (40.9) | 789 (40.3) |
| 20-<30 ng/ml: Sufficiency | 39 (35.8) | 37 (19.0) | 90 (40.5) | 106 (25.4) | 119 (26.6) | 129 (22.8) | 272 (28.8) | 248 (24.5) | 520 (26.6) |
| ≥30-80 ng/ml: Physiological status^2)^ | 9 (8.3) | 9 (4.6) | 32 (14.4) | 51 (12.2) | 94 (21.0) | 43 (7.6) | 101 (10.7) | 137 (13.5) | 238 (12.2) |

1. criteria for the classification stages: see “Method” section
2. the highest 25 OH D level was 75 ng/ml
