# Supplementary material for: No improvement in vitamin D status in German infants and adolescents between 2009 and 2014 despite public recommendations to increase vitamin D intake in 2012
Source: Eur J Nutr. 2018 May 18;58(4):1711–22. doi: 10.1007/s00394-018-1717-y (PMC6561984; doi:10.1007/s00394-018-1717-y)
Supplement: Supplementary file 2 — Supplementary material 2 (DOCX 15 KB) [file 394_2018_1717_MOESM2_ESM.docx]

**Suppl. Table 2** Distribution of serum 25(OH)D levels by age class according to the four classification stages^1)^

|  |  |  | Age class |  |  |  |
| --- | --- | --- | --- | --- | --- | --- |
|  | 0–2 years | 3–6 years | 7–10 years | 11–13 years | 14–17 years | Total |
| Subjects with |  |  | **n (%)** |  |  |  |
| **Deficiency (<20 ng/ml)** | **68 (54.0)** | **205 (62.7)** | **318 (61.1)** | **325 (61.6)** | **283 (62.0)** | **1199 (61.3)** |
| <10 ng/ml: Severe deficiency | 18 (14.3) | 63 (19.3) | 112 (21.5) | 106 (20.1) | 111 (24.3) | 410 (21.0) |
| 10-<20 ng/ml: Deficiency | 50 (39.7) | 142 (43.4) | 206 (39.6) | 219 (41.5) | 172 (37.7) | 789 (40.3) |
| 20-<30 ng/ml: Sufficiency | 36 (28.6) | 88 (26.9) | 135 (26.0) | 144 (27.3) | 117 (25.7) | 520 (26.6) |
| ≥30-80 ng/ml: Physiological status^2)^ | 22 (17.5) | 34 (10.4) | 67 (12.9) | 59 (11.2) | 56 (12.3) | 238 (12.2) |

1. criteria for the classification stages: see “Method” section
2. the highest 25(OH)D level was 75 ng/ml
